# Phytochemical profile and antioxidant activity of various solvent extracts of two varieties of ginger and garlic

**DOI:** 10.1016/j.heliyon.2023.e18806

**Published:** 2023-07-30

**Authors:** Jolly Oder Akullo, Beatrice N. Kiage-Mokua, Dorothy Nakimbugwe, Jeremiah Ng’ang’a, John Kinyuru

**Affiliations:** a Department of Animal Production and Management, Faculty of Agriculture and Animal Sciences, Busitema University, Uganda; b Department of Human Nutrition Sciences, School of Food and Nutrition Sciences, Jomo Kenyatta University of Agriculture and Technology, Kenya; c Department of Food Technology and Nutrition, School of Food Technology, Nutrition and Bio-engineering, Makerere University, Uganda; d Department of Food Science and Technology, School of Food and Nutrition Sciences, Jomo Kenyatta University of Agriculture and Technology, Kenya; e African Institute for Capacity Development, P.O. Box 46179 – 00100, Nairobi, Kenya

**Keywords:** Phenolic, Flavonoids, Inhibitory concentration, Natural antioxidants

## Abstract

There is limited information on the phytochemical profile and antioxidant activity of ginger and garlic consumed in Uganda. This could have an impact on its widespread use and industrial application. Thus, this study was done to determine the phytochemical profile and antioxidant activity of two varieties of ginger and garlic commonly consumed in Uganda. Fresh ginger rhizomes and garlic cloves of "local" and "hybrid" varieties were acquired from a local food market, washed, grated, and extracted using acetone, ethanol, methanol, and water. Standard techniques were used to determine the phytochemical composition. Total phenolic and flavonoid content were measured using Folin-Ciocalteu and aluminium chloride assays, respectively. Antioxidant activity was determined using the 2, 2-Diphenyl-1-picryl hydrazyl (DPPH) assays. Ginger extracts exhibited significantly higher total phenolic and flavonoid content compared to garlic (p˂0.05). The highest total phenolic and flavonoid content was in ethanol and methanol extracts of local ginger: 1968.49 and 2172.65 mg GAE/100 g; 254.24 and 184.62 mg QE/100 g, respectively. Tannins, alkaloids, saponins, and terpenoids were in varying concentrations in the extracts. Levels of Vitamin C were significantly high in aqueous extracts (p˂0.05), 38.34 and 40.80 AAE/100 g in local and hybrid ginger; 33.65 and 35.24 mg AAE/100 g in local and hybrid garlic, respectively. The free radical scavenging activity of extracts varied depending on concentration, with a strong positive correlation between antioxidant activity and total phenolic and flavonoid content. The half maximal inhibitory concentration (IC50) ranged from 0.16 to 8.93 mg/ml in local ginger, 4.43–6.44 mg/ml in hybrid ginger, 3.93–5.64 mg/ml in local garlic, and 4.44–5.27 mg/ml in hybrid garlic. The best antioxidant activity was exhibited by ethanol extracts of the local ginger. According to the findings, the two varieties of ginger and garlic have strong antioxidant activity due to their different phytochemical compositions, which could make them useful as natural antioxidants in food and medicine applications.

## 1 Introduction

Herbs and spices have been used for centuries to enhance the flavor of food, preserve food from spoiling during storage, and treat or prevent human ailments [1, 2]. Herbs and spices are often considered safe for human consumption compared to conventional treatments because of their long history of use in food preparation [3, 4]. They are utilized in food either in their natural state (whole or ground materials) or as extracts, which is more common [5].

Ginger and garlic have long been recognized as health-promoting agents, and their extracts have been studied as alternative remedies for a variety of conditions, including gastric-intestinal and oral cavity diseases, cough, and arthritis among others. These spices are also commonly utilized in pharmaceutical products [1, 6, 7]. In an *in vivo* study, a combined extract of ginger and garlic exhibited anti-inflammatory, anti-oxidant, anti-lipidemic, and immunomodulatory activities [8].) The high antioxidant capacity of ginger and garlic is attributed to their various phytochemical components, which are responsible for relieving oxidative stress [9].

Generally, the quality and quantity of plant extracts, and their effectiveness as antioxidant agents, are influenced by the characteristics of the original plant material, the extraction technique, and the extraction solvent, among other factors [10]. Although several unique extraction techniques have recently been developed, conventional methods using solvent extraction remain the most widely used method for extracting phytochemicals. The majority of the phytochemicals in plants, including phenolics, flavonoids, and anthocyanins, are extracted using polar and medium polar solvents such as; water, ethanol, methanol, propanol, acetone, and their aqueous mixtures [11]. The advantages of conventional methods of extraction are that they are s simple to use, have a wide range of applications, and do not necessitate sophisticated equipment [12]. However, from an environmental aspect, solvent extraction is not a green chemistry approach because it generates a large volume of waste with possibly hazardous residues depending on the type of solvent used [13, 14]. The selection of an optimal solvent that maximizes the profile and quantity of bioactive chemicals eluted, thereby increasing bioactivity, is a critical stage in the extraction process. A study on phytochemical screening and antioxidant activities of red button ginger (Costus woodsonii) recommended using sequential extraction with solvents of different polarities to elute the maximum amount of compounds present in this plant [15].

Ginger (Zingiber officinale) belongs to the Zingiberaceae family. It is grown and used as a spice and medicinal herb in the tropics of Asia, Africa, America, and Australia [16]. More than 150 zingiberaceous species have been identified in the wild and in cultivation [17]. Cultivated ginger has a wide range of rhizomes and vegetative features, according to research, and environmental factors have a significant impact on its important bioactive compounds [18]. Garlic (*Allium sativum*) is a perennial, bulbous herb belonging to the Alliaceae family. There are two types of garlic: hardneck and softneck [19]. The center stalks of hardneck garlic are hard, woody, and extend all the way down to the bulb's basal plate. Soft-necked garlic has a non-woody pseudostem made up of overlapping leaf sheaths, and it rarely produces a flower stalk unless the environment is stressful. It is thought that garlic with a soft neck originated from garlic with a hard neck [19]. review the genetic diversity of the garlic classification.

In Uganda, two species of ginger and garlic are extensively used as medicinal plants and condiments in food preparation to improve the flavor and aroma as well as the keeping quality [20, 21]. "Local" ginger and "hybrid" ginger, as well as "local" garlic and "hybrid" garlic, are terms used by indigenous people (traders and consumers). The term "local" ginger refers to a ginger cultivar with short rhizomes, thick brown skin, yellow flesh or powder, and a pungent scent. The variety with big rhizomes, light yellow skin, off-white flesh or powder, and a less pungent aroma is referred to as "hybrid" ginger. "Local" garlic, on the other hand, refers to a locally grown type with small bulbs, purple skin, and woody stalks that is typically grown in the coldest parts of the country. While the "hybrid" garlic, also known as "Chinese garlic," has large bulbs, silver-white bulb scales, and a tender feel and according to traders, “hybrid garlic” is not grown in the country but is imported from China and has a longer shelf life. The hard neck and soft neck classifications of garlic types correlate to the characteristics of local and hybrid garlic varieties, respectively [19]. Soft neck garlic, as opposed to hard neck garlic, have a higher number of protective shells and thus a longer shelf life [22].

The phytochemical content and antioxidant activity of 16 ginger-like plants (Zingiberaceae family) extracted using n-hexane, ethyl acetate, and ethanol were examined [23]. The results demonstrated that the antioxidant capabilities of ethyl acetate and ethanol extracted samples were superior to those of n-hexane extracts. According to Ref. [24]; two Malaysian ginger varieties extracted with ethanol had different phytochemical compositions and antioxidant activities [25]. determined the chemical composition and antioxidant activity of aqueous and solvent extracts of ginger root (*Zingiber officinale*); the results showed that phytochemical (polyphenols, flavonoids, and total tannin) concentrations were higher in hot water (100 °C) extract than other solvent extracts, but antioxidant activity was higher in solvent extract than water extract using three different methods. Ethanol extracts from the rhizomes of five ginger plants were reported to possess antioxidant and anti-acetylcholinesterase properties, which were attributed to the presence of polyphenols and flavonoids in the plants [26]. The main bioactive compounds in ginger are gingerols, shogaols, and paradols, with gingerols being the most abundant polyphenol in fresh ginger [27].

Total phenolic and flavonoid contents and five antioxidant parameters, including free radical scavenging ability, were measured in 43 garlic cultivars. The results showed variations among the garlic cultivars, and the antioxidant properties were highly correlated with the presence of the primary phenolic compounds [28]. The bioactivity of garlic is attributed to the presence of sulphur containing compounds such as allicin and allin [29].

[7] investigated the efficacy of different organic solvents (80% acetone, 80% ethanol, 80% methanol) and distilled water in extracting antioxidant phenolic components from turmeric, curry leaf, torch ginger, and lemon grass extracts. The most efficient solvent was identified to be 80% ethanol, and the amount of phenolic compounds varied based on the solvents utilized [10]. investigated the effects of solvent and duration of extraction on the antioxidant properties and phenolic profiles of 13 herbs and spices used for food seasoning and preservation. Long-time extraction (24 h) with 50% aqueous ethanol was found to be the best extraction condition for obtaining the highest total phenolic and antioxidant activity.

Even though numerous studies have reported the phytochemicals and antioxidant activities of ginger and garlic, there is limited information on the phytochemical profile and antioxidant activity of ginger and garlic varieties commonly consumed in Uganda, let alone the effect of the extraction solvent on phytochemical composition and antioxidant activity. This could have an impact on its widespread use and industrial application. As a result, the current study used various solvent extraction systems to investigate the phytochemical composition and antioxidant activities of ginger and garlic cultivars commonly used in Uganda.

## 2 Material and methods

### 2.1 Collection and preparation of samples

Freshly harvested ginger rhizomes and garlic cloves (2.5 kg) each were acquired from a local food market in Northern Uganda (2.2581° N, 32.8874° E), packed in airtight bags, and transported to the Jomo Kenyatta University of Agriculture and Technology (JKUAT) food biochemistry laboratory in Kenya. Samples were carefully cleaned under tap water, drained to eliminate excess water, and grated for extraction in the laboratory.

### 2.2 Phytochemical extraction and sample preparation for antioxidant assays

With minor adjustments, samples were extracted using absolute (99.9%) acetone, ethanol, methanol, and distilled water at room temperature (23 ± 2 °C) according to the technique of [30]; 2 g of freshly grated ginger and garlic were placed in each of the 100 ml amber bottles along with 30 ml of a specific solvent; 30 ml of distilled water was also used to make aqueous extracts. Both the organic and aqueous mixtures were shaken for 1 h at 300 rpm in a mechanical shaker (Labortechnik KS 250b, Germany) and then kept in the dark for 72 h to avoid the flask contents reacting with light. After that, the extracts were filtered using Whatman filter paper No. 1 and tested for various phytochemical components and antioxidant activity, as described below.

### 2.3 Phytochemical analysis

#### 2.3.1 Determination of total phenolic concentration

The total phenolic content (TPC) of ginger and garlic extracts was determined using the Folin-Ciocalteu method, according to previously described procedures [31]. Precisely, 10 mg of gallic acid was dissolved in 100 ml of 50% methanol and diluted to 10, 20, 30, 40, 50, and 60 g/ml. An aliquot (1 ml) from each dilution was transferred to a test tube and diluted with 10 ml of distilled water. Then 2 ml of Folin-Ciocalteu's reagent was added, vortexed, and left to incubate for 5 min at room temperature. In each test tube, 4 ml of 0.7 M Na_2_CO_3_ were added, adjusted with distilled water up to the mark of 25 ml, vortexed, and held for 30 min at room temperature. The absorbance of the standard was measured against a blank using a UV/VIS spectrophotometer (Shimadzu model UV-1601 PC, Kyoto, Japan) (distilled water). Similarly, 1 ml of the sample extracts were prepared and analyzed using a comparable process. A standard curve was then plotted using garlic acid, and total phenolic contents were calculated using the linear equation on the standard curve. Total phenolic components were calculated as mg/100 g extract of gallic acid equivalent (GAE).

#### 2.3.2 Determination of flavonoid concentration

Flavonoids were determined using the aluminum chloride colorimetric technique [32]; distilled water (4 ml) and 1 ml of ginger and garlic extracts were added to a 10 ml volumetric flask; after 3 min, 0.3 ml of 5% sodium nitrite solution was added. Then, 0.3 ml of 10% aluminum chloride was added after 3 min; 2 ml of 1 M sodium hydroxide was added after 5 min; and the volume was brought up to 10 ml with distilled water. A UV–Vis spectrophotometer was used to detect absorbance at 415 nm (Shimadzu model UV-1601 PC, Kyoto, Japan). Quantitative determination of total flavonoids was done on the basis of a standard curve of quercetin and linearity of the calibration curve was achieved between 0 and 1000 μg/ml concentration for quercetin (r^2^ = 0.9965). Total flavonoids were reported in mg quercetin equivalent (QE)/100 g of ginger or garlic extract.

#### 2.3.3 Determination of tannin concentration

Tannins were measured using a modified vanillin-hydrochloric acid method [33]. Catechin hydrate was used to make standards at concentrations of 0, 10, 20, 40, 60, 80, and 100 mg/ml (r^2^ = 0.9987). Duplicate aliquots of 1 mL of each sample extract were placed in test tubes, one of which acted as a sample blank; to the blanks, 5 mL of 4% HCl in methanol without the reagent (vanillin) was added. Samples and standards, on the other hand, were treated for 20 min with 5 ml of vanillin-HCl reagent (made by mixing equal quantities of 8% HCl in methanol and 1% vanillin in methanol right before use). At 500 nm, the absorbance of the standards, samples, and blanks was measured, and tannin content was estimated as percent catechin equivalent (CE) using a standard calibration curve derived from catechin absorbance at various concentrations.

#### 2.3.4 Determination of ascorbic acid concentration

The HPLC technique was used to determine the ascorbic acid content of the samples [34]. The solution was centrifuged at 10,000 rpm at 4 °C after extracts from 2 g of sample were combined with 0.8% metaphosphoric acid. The supernatant was filtered, and 10 mL of 0.8% metaphosphoric acid was added to it. This was filtered using a 0.45 μl filter and 20 μl injected into the high-performance liquid chromatography (HPLC) machine. The Shimadzu UV-VIS detector was used for HPLC analysis; at a flow rate of 1.2 ml/min and a wavelength of 266.0 nm, the mobile phase was 0.8% metaphosphoric acid. A calibration curve was generated from several concentrations (0, 20, 40, 60, 80, 100 μg/ml) of ascorbic acid standards (r^2^ = 0.9881). Ascorbic acid content was reported in mg of ascorbic acid equivalent (AAE)/100 g of ginger or garlic extracts.

#### 2.3.5 Determination of alkaloids

The alkaloid content was determined using the procedures of [31]; with some variations. The extract from 2 g of sample was concentrated to one quarter of its original volume in a water bath (50 °C) for 4 h. Dropwise additions of absolute ammonium hydroxide (10 ml) to the concentration were made until the precipitation was complete. The precipitate was collected, rinsed with dilute (2 M) ammonium hydroxide, and filtered after the solution had settled. After that, the residue was classified as alkaloids and was dried and weighed. The proportion of alkaloids in the sample was calculated using the formula below.

$$Alkaloid\;\left(\%\right)=\left(\frac{W3-W2}{W1}\right)*100$$

Where; *W3* = weight of sample residue before drying; *W2* = weight of residue after drying; *W1* = original sample weight.

#### 2.3.6 Terpenoids and saponin screening

The Salkowski test was used to determine terpenoids qualitatively, following established technique [35]. Each ginger and garlic extract (5 ml) was carefully combined with 2 ml of 100% chloroform (v/v) and absolute H_2_SO_4_ (3 ml) to produce a layer. The presence of terpenoids was revealed by the formation of a reddish-brown layer at the contact. The basic foam test was used to determine the amount of saponin [36]. Extracts (5 ml each) was transferred to separate test tubes and diluted with 5 ml of distilled water; the mixture was firmly agitated for 2 min. The presence of foam that lasted at least 15 min confirmed its presence.

### 2.4 Antioxidant assays of ginger and garlic extracts

The antioxidant activity (free radical scavenging ability) of organic solvent and aqueous extracts of ginger and garlic was evaluated in vitro using 2,2-diphenyl-1-picryl hydrazyl (DPPH) radical (Sigma-Aldrich, USA) assay, following the procedure of [37]; with modifications. DPPH is a stable free radical that generates a deep purple color in solution. When DPPH accepts an electron from an antioxidant, it becomes colorless or pale yellow. This neutralization process can be measured spectrophotometrically by the changes in absorbance at 517 nm. In a study by Ref. [28] the DPPH assays of spice extracts showed a significant correlation to other methods of antioxidant evaluation, thereby demonstrating their reliability. The extracts were produced at concentrations of 0, 1, 2, 4, 6, 8, and 10 mg/ml in methanol (analytical grade). In the same quantities as the extract, vitamin C was utilized as an antioxidant standard. In a test tube, 1 ml of the extract was added to 3 ml of methanol, followed by 0.5 ml of 1 mM DPPH in methanol. A control sample was made with the same amount of methanol and DPPH. Methanol was used to zero the spectrophotometer, and the absorbances were read at 517 nm after 5 min. The radical scavenging activity was calculated using the following formula:

$$\%\;inhibition\;of\;DPPH=\left(\frac{AB-AA}{AB}\right)*100$$

Where AB is the absorbance of control sample and AA is the absorbance of tested extract solution.

The results were expressed as percentage inhibition of DPPH and minimum inhibitory concentrations (IC50), determined using a linear equation from a plot of % inhibition of DPPH versus concentration. The IC50 value is a parameter widely used to measure the antioxidant activity of test samples. It is calculated as the concentration of antioxidants needed to decrease the initial DPPH concentration by 50%. Thus, a lower IC50 value indicates higher antioxidant activity.

### 2.5 Data analysis

All analysis were done in triplicates and values were reported as mean ± standard deviation (SD). ANOVA tests were performed using GenStat software to determine significant differences among extracts. Statistical comparisons were separated using the Bonferroni post hoc test with the Least Significant Differences (LSD) considered at P ≤ 0.05.

## 3 Results and discussion

### 3.1 Total phenolic, flavonoid and tannin content of ginger and garlic extracts

The phenolic content of aqueous and organic solvent extracts of hybrid and local ginger differed significantly (p˂0.05); from 285 to 2172 mg GAE/100 g in water and methanol extracts of the local ginger, respectively (Table 1). TheTPC of ethanol extracts of hybrid and local ginger (1595 and 1968 mg GAE/100 g, respectively) was higher than reported for three Malaysian ginger varieties, which had 1053, 1127, and 1276 mg GAE/100 g [ [38,39]]. The TPC of organic solvent extracts of ginger in this study was higher than previously reported for acetone and methanol extracts of ginger (1093.4 and 871.5 mg/100 g, respectively) [16]. In a study by Ref. [40]; the highest total phenolic content was obtained from the chloroform/methanol extract of fresh ginger rhizomes (60.34 mg GAE/g), when compared to pure chloroform or methanol extracts.

**Table 1:** Total phenolic, flavonoid and tannin content of hybrid and local varieties of ginger and garlic extracted with different solvents.

Type	Solvent	Total Phenolic (mg GAE/100 g)	Total Flavonoids (mg QE/100 g)	Tannins (mg CE/100 g)
Hybrid ginger	Acetone	1217.85 ± 76.31^b^	168.52 ± 6.02^bc^	35.08 ± 5.45^bc^
	Ethanol	1595.98 ± 67.80^c^	205.86 ± 24.30^d^	33.13 ± 0.94^bc^
	Methanol	1322.05 ± 17.74^b^	148.33 ± 6.36^b^	55.47 ± 5.84^d^
	Water	385.13 ± 36.94^a^	104.50 ± 4.00^a^	3.69 ± 1.82^a^
Local ginger	Acetone	1278.85 ± 40.78^b^	168.52 ± 16.70^bc^	28.12 ± 1.45^b^
	Ethanol	1968.49 ± 50.72^d^	254.24 ± 14.13^e^	42.36 ± 2.50^c^
	Methanol	2172.65 ± 13.10^d^	184.62 ± 10.71^cd^	69.43 ± 7.80^e^
	Water	285.66 ± 42.47^a^	107.18 ± 4.20^a^	3.788 ± 0.45^a^
***P- value***		***<0.001***	***0.002***	***0.002***
Hybrid garlic	Acetone	164.86 ± 26.74^cde^	75.33 ± 5.84^b^	7.44 ± 2.19^ab^
	Ethanol	207.19 ± 12.75^e^	124.33 ± 3.71^c^	27.31 ± 3.70^c^
	Methanol	118.99 ± 12.18^bc^	64.68 ± 5.57^b^	16.84 ± 1.63^abc^
	Water	40.87 ± 2.63^a^	23.78 ± 1.52^a^	3.80 ± 1.73 ^ab^
Local garlic	Acetone	172.01 ± 16.32^cde^	64.84 ± 1.93^b^	6.52 ± 1.07^ab^
	Ethanol	147.52 ± 13.83^cd^	88.40 ± 0.69^b^	22.26 ± 4.45^c^
	Methanol	191.04 ± 36.60^de^	84.50 ± 5.90^b^	17.45 ± 2.19^bc^
	Water	64.89 ± 3.61^ab^	26.78 ± 2.72^a^	3.14 ± 0.78^a^
		***0.177***	***<.001***	***0.696***

Values = Mean ± SD (n = 3). Values with different superscripts along the column differ significantly for each spice.

The total phenolic content of the water extract was low, with 285 mg GAE/100 g in local ginger and 385 mg GAE/100 g in hybrid ginger, respectively, which was comparable to the 297.88 mg GAE/100 g reported for ginger rhizomes from China [41]. As a result, the findings show that organic solvents are better for polyphenol extraction than water. The type of solvent used in quantitative extraction is important, and 60–80% ethanol in water has been identified as the most promising solvent for most phenolic groups [12]. This is in line with prior studies that showed polyphenols to have poor water solubility [13].

On the other hand, organic solvent extracts of garlic had a higher phenolic content than aqueous extracts, and ethanol extracts of hybrid garlic had a much higher phenolic content than local garlic (Table 1). The phenolic content of garlic extracts in this study (40.87–207.2 mg GAE/100 g) was higher than the 42.6 mg GAE/100 g reported in an acidified methanol extract of garlic from Poland [42], but lower than the range of 17.17–42.53 mg GAE/g reported for 43 garlic cultivars in China [28].

According to Ref. [43]; total phenolic content in garlic varieties ranged from 3.4 to 10.8 mg GAE/g, with white garlic cultivars and Chinese garlic cultivars having higher total phenolic content than purple garlic cultivars. The findings show how different varieties affect total phenolic content. The presence of OH groups in the chemical structure of phenolic compounds confers antioxidant capabilities. Therefore, the total phenolic content has been used to screen for antioxidant activity in plants [23,25].

Flavonoids are polyphenolic secondary metabolic compounds with a low molecular weight that are found in all plants. Flavonone, flavone, isoflavone, flavonol, catechin, naringin, and anthocyanins are classes of flavonoids [44]. The highest TFC was recorded in ethanolic extracts; 254.2 and 205.9 mg QE/100 g in local and hybrid ginger; 124.33 and 88.40 mg QE/100 g in hybrid and local garlic, respectively. However, in both ginger and garlic varieties, organic solvent extracts had significantly higher TFC compared to the aqueous extracts (p˂0.05) (Table 1). The TFC in this study were comparable to those found in a variety of spices [10]. The values observed for ginger (107.2–254.2 mg QE/100 g) were lower than previously reported: 379 and 428 mg QE/100 g for ethanol extracts of ginger cultivars in Malaysia [38]; 655 and 4025 mg QE/100 g for chloroform/methanol and petroleum ether extracts of Sudanese ginger rhizomes [40].

In a previous study of 43 Chinese garlic cultivars, the TFC ranged from 0.15 to 0.60 mg rutin DW/g [28]. Extracts with high flavonoid content also had a high total phenolic content. The solubility of flavonoids in various solvents is known to vary, and the choice of its extraction solvent is frequently dependent on polarity. Variation in flavonoid concentration could be due to the intrinsic variability in plant materials. High flavonoid content suggests the usefulness of the plant material since flavonoids have become an essential component in a wide range of nutraceutical, pharmacological, therapeutic, and cosmetic applications [44]. Also, flavonoids are highly effective scavengers of most oxidizing molecules, including singlet oxygen and various free radicals [45]. The antioxidant capacity of flavonoids varies depending on the type of functional group and how it is arranged around the nuclear structure [46].

The tannin concentration in ginger extracts was significantly different (p˂ 0.05); ranging from 3.79 to 69.43 mg CE/100 g in water and methanolic extracts of the local ginger. However, the tannin content of local and hybrid ginger extracts in ethanol, acetone, and aqueous extracts did not differ significantly. Similarly, the tannin content of organic and aqueous extracts of both varieties did not differ significantly (Table 1). Methanolic extracts of local and hybrid ginger had the highest tannin content (69.43 and 55.47 mg CE/100 g, respectively), whereas water extracts had the lowest. Ethanolic extracts of hybrid and local garlic had the highest tannin concentrations, at 22.26 and 27.31 mg CE/100 g, respectively. This implies that the most effective solvents for extracting tannins from ginger and garlic are methanol and ethanol, respectively. This is consistent with a recent study that found that tannin concentrations in methanol extracts were significantly greater than in water extracts [47].

Tannin content was found to be lower in this study than previously reported for spices [48,49]. However, when the effects of extraction solvents were compared, it was revealed that ethanol and methanol extracted tannins more effectively than acetone [25]. Tannins have hydroxyl groups at various positions in their structure, as well as other functional groups like carboxyl, which are critical for the formation of complexes with macromolecules and proteins, and hence determine their solubility [50].

Recently, researchers have become more interested in employing tannin-rich plants and plant extracts in the diets of ruminant animals in order to improve the quality of the resultant products [51]. In several food products, plant tannins have been shown to have antioxidant effects. A study in mutton revealed that tannin supplementation increased the color stability of the *longissimus dorsi* muscle (LM), with less variations in the hue angle in the treatment groups than in the control groups [52].

### 3.2 Vitamin C, alkaloids, saponins and terpenoids in ginger and garlic extracts

Levels of vitamin C varied significantly among ginger and garlic extracts (p˂0.05); being highest in aqueous extracts (40.80 and 35.24 mg/100 g) of local ginger and hybrid garlic, respectively (Table 2). The high levels of vitamin C in water extracts are expected as it is a water-soluble vitamin. Ascorbic acid is a polar organic molecule that has many hydroxyl groups in its structure. For that reason, it is believed that smaller molecules such as water compared to ethanol and other organic solvents achieve equilibrium more easily because the hydroxyl groups on vitamin C establish hydrogen bonds, favoring the increase of its solubility [53]. Efficient water extraction of phytochemicals is an interesting result due to the general safety of extracts in food applications and the low cost of extraction.

**Table 2:** Vitamin C, alkaloids, Saponin and terpenoids of fresh ginger and garlic extracted using different solvents.

Spice Variety	Solvent	Vitamin C (mg AAE/100 g)	Alkaloid (%)	Saponins	Terpenoids
Hybrid ginger	Acetone	8.00 ± 0.13^a^	12.37 ± 0.89^c^	++	+
	Ethanol	18.33 ± 0.16^d^	13.05 ± 1.19^c^	++	++
	Methanol	15.98 ± 0.07^b^	9.35 ± 0.66^ab^	+	++
	Water	40.80 ± 0.12^f^	13.15 ± 1.09^c^	++	+
Local ginger	Acetone	7.51 ± 0.07^a^	9.27 ± 0.01^ab^	++	+++
	Ethanol	17.25 ± 0.27^c^	10.37 ± 0.27^b^	+	+++
	Methanol	15.52 ± 0.63^b^	8.80 ± 0.95^a^	+	+++
	Water	38.34 ± 0.75^e^	12.68 ± 0.27^c^	+	+++
***pvalue***		***<.001***	***0.009***		
Hybrid garlic	Acetone	10.29 ± 0.06^b^	6.66 ± 0.75^a^	ND	+++
	Ethanol	13.95 ± 0.11^d^	11.48 ± 0.29^c^	ND	+++
	Methanol	16.58 ± 0.08^f^	10.90 ± 0.27^bc^	+	+
	Water	35.24 ± 0.05^h^	13.02 ± 0.60^d^	+	++
Local garlic	Acetone	9.22 ± 0.11^a^	6.30 ± 0.74^a^	+	+++
	Ethanol	13.54 ± 0.67^c^	10.42 ± 0.36^b^	+	+
	Methanol	15.65 ± 0.02^e^	11.00 ± 0.57^bc^	+	+
	Water	33.65 ± 0.04^g^	12.67 ± 0.67^d^	++	+
***p***		***<.001***	***0.360***		

Values = Mean ± SD (n = 3). Values with different superscripts in the column differ significantly for the variety. Key +: present in minute quantity, ++: present in medium quantity, +++: present in large quantity, ND: Not Detected.

Among the organic solvent extracts, ethanolic extracts of hybrid ginger had a higher vitamin C content than local ginger, while methanolic extracts of hybrid garlic had a higher vitamin C content than local garlic. This indicates that the vitamin C concentration of extracts is influenced by both the solvent and variety, which is consistent with prior results on the vitamin C content of fruits and vegetables [54]. Levels of vitamin C in this study were comparable to previous reports for spices in Ghana [55].

The levels of vitamin C in aqueous extracts of local and hybrid ginger (38.34 and 40.80 mg/100 g, respectively) was comparable to the 48 mg/100 g reported for ginger rhizomes from India [56]. In another study, a range of 4.44–31.51 mg/100 g was reported for methanolic extracts of six varieties of garlic produced and harvested under identical conditions [57]. Vitamin C is a potent antioxidant that interacts directly with a wide spectrum of harmful reactive oxygen species via electron transfer to inhibit the reaction initiated by free radicals; it also contributes to the regeneration of other antioxidants, such as tocopherol, to their functioning state [56,58].

The highest alkaloid content was observed in the ethanol extract of the hybrid ginger, (13.05%) which was statistically comparable to the aqueous extracts of both varieties and the acetone extract of the hybrid ginger (Table 2). Methanol extracts of the local (8.80%) and hybrid ginger (9.35%) had the lowest quantity of alkaloids. Aqueous extracts of garlic had a statistically similar high alkaloid quantity; (12.67 and 13.02%) in local and hybrid garlic; the alkaloid concentration was lowest in acetone extracts of local and hybrid garlic (6.30 and 6.66%, respectively). Alkaloid concentration was in the range reported for other spices: pepper (13.44%), ginger (11.21%), and 2.54% in garlic [59]; 15.38 and 11.32% in ginger and garlic [60]; 0.15 and 0.012 mg AE/g for ginger and garlic [61]. Variations are attributed to the difference in the method of testing, as the previous study employed a spectrophotometric method. Previous screening of phytochemicals in herbs and spices did not detect alkaloids in extracts of ginger (Z. officinale) and garlic (A. sativum) obtained from Nigeria [62].

Quantitatively, moderate amounts of saponins were detected in acetone extracts of hybrid and local ginger and in ethanolic and aqueous extracts of the hybrid ginger. Whereas, saponins were not detected in acetone and ethanol extracts of the hybrid garlic. In a previous study, absolute methanol, ethanol extracts, and hot water extracts of ginger contained saponins; organic solvent extracts of garlic contained saponins; while the hot water extracts did not contain saponins [60]. This is contrary to previous reports in which moderate quantities were reported for ginger while high quantities were reported for garlic extracts [62]. Saponins are characterized by surface-active foaming properties, a bitter taste, and astringency. It is reported to have various health benefits, including medicinal and pharmacological applications, due to its foaming ability and the production of frothy effects [63].

On the other hand, large quantities of terpenoids were present in all the organic and aqueous solvent extracts of the local ginger, while minute quantities were present in the acetone and aqueous extracts of the hybrid ginger. In garlic, acetone extracts of both varieties and ethanol extracts of hybrid garlic contained large quantities of terpenoids. In a previous study, terpenoids were detected in both organic solvent and aqueous extracts of ginger and garlic [60]. However, terpenoids concentration was higher in garlic than in ginger extracts [64,61]. Previous research has shown that terpenoids have anticancer, antibacterial, antifungal, antimalarial, and anti-inflammatory properties [65].

### 3.3 Antioxidant activity of ginger and garlic extracts

#### 3.3.1 DPPH free radical scavenging ability of ginger extracts

**Fig. 1:**
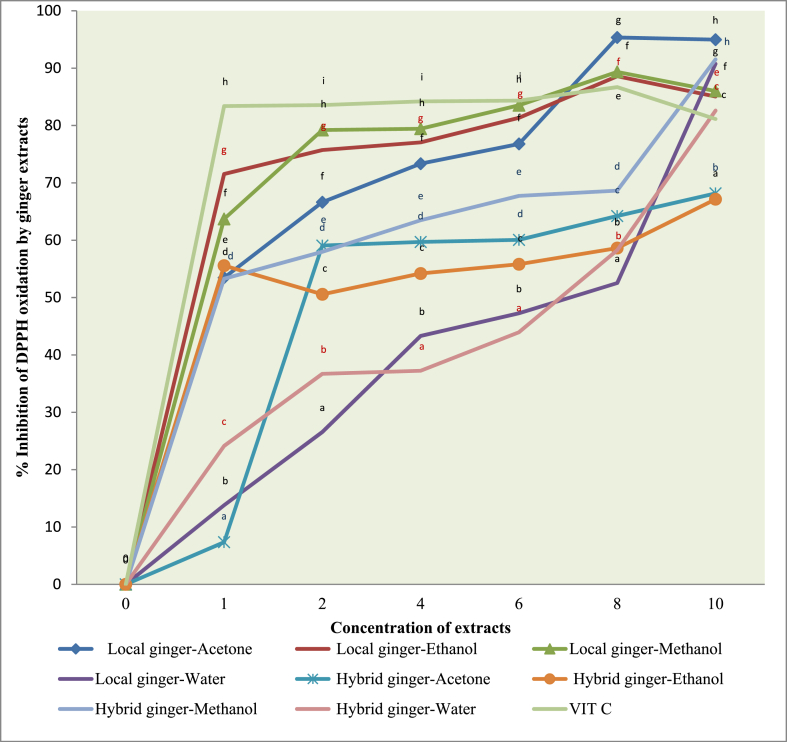
DPPH free radical scavenging ability of Ginger extracts in varying concentration. Values plotted is the mean of triplicate determination, different superscripts at the same concentration denote significant difference in inhibition percentage (p˂0.05).

The results of this study showed that all extracts exhibited free radical scavenging activity in a dose-dependent manner from 1 to 10 mg/ml. The organic extracts of the local ginger had a higher DPPH free radical scavenging activity compared to the aqueous extracts (Fig. 1). Ethanol and methanol extracts of the local ginger had the highest DPPH inhibition: 71.54% and 63.7% at 1 mg/ml, and 85.05% and 86.01% at 10 mg/ml, respectively. The inhibition potential of the ethanol and methanol extracts of the local ginger compared favorably with the standard (vitamin C) across all concentrations: 83.38% at 1 mg/ml and 81.14% at 10 mg/ml.

The organic extracts of the hybrid ginger exhibited moderate inhibition of the DPPH free radical at most concentrations in comparison to the local ginger, while the water extracts of the local and hybrid ginger exhibited the lowest free radical scavenging activity at most concentrations. The inhibition of ethanol and methanol extracts of hybrid ginger (55.57% and 53.29%) at 1 mg/ml was lower than that of ethanolic extracts (66.04%) of ginger powder [9]; and higher than reported (41.7% and 49.7%) for ethanolic extracts of fresh rhizomes of ginger varieties in Malaysia [38]. Meanwhile, 32.7, 27.0, 28.4, and 11.5% were reported for acetone, ethanol, methanol, and aqueous extracts of torch ginger [7]. This is consistent with a similar study showing differences in DPPH percentage inhibition and IC50 of various solvent extracts of 16 ginger-like plants [23].

**Fig. 2:**
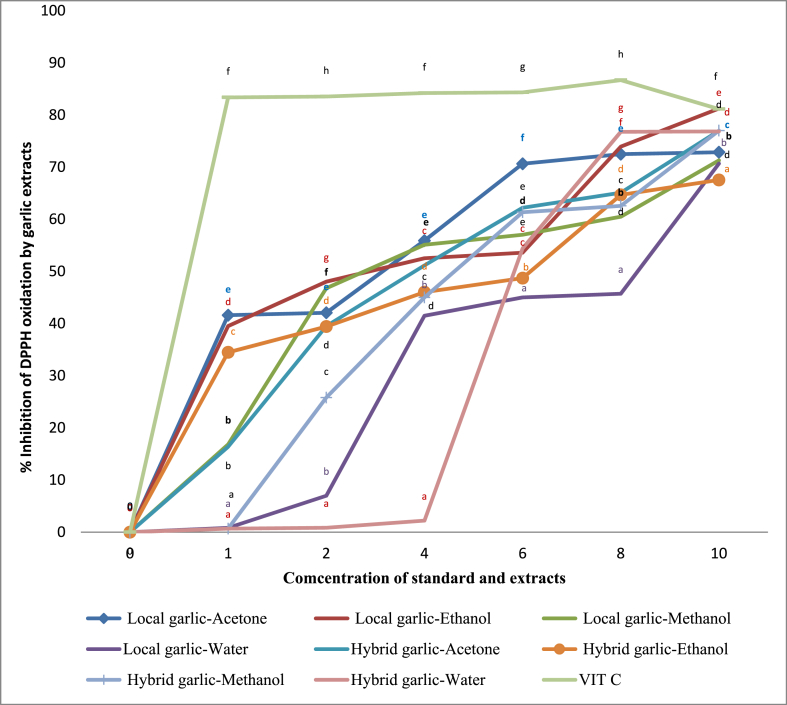
DPPH free radical scavenging ability of Ginger extracts in varying concentration. Values plotted is the mean of triplicate determination, different superscripts at the same concentration denote significant difference in inhibition percentage.

In garlic extracts, DPPH free radical scavenging activity was significantly different and lower than the standard (P˂ 0.05). At most concentrations, the free radical scavenging ability of organic extracts was stronger than that of water extracts in both local and hybrid garlic (Fig. 2). Ethanol and acetone extracts of the local garlic had higher inhibition at 1 mg/ml (39.53% and 41.61%) and at 10 mg/ml (81.24% and 72.86 compared to the hybrid garlic. These results were within the range reported for garlic water soluble components and garlic oil [66]; though lower than the 97.95% reported in aged garlic [67]. This is attributed to the nature of the products, which differ in that aged garlic extract (AGE) is made by soaking fresh garlic in aqueous ethanol and maturing it for several months. Our findings are consistent with research that shows different garlic cultivars have variable levels of free radical scavenging abilities [68]; a range of 3.60%–45.63% was reported for 43 varieties of garlic [28].

#### 3.3.2 IC50 of ginger and garlic extracts

A significant variation existed in the IC50 of local and hybrid ginger extracted with organic and aqueous solvents (p˂0.05). The half maximal inhibitory concentration (IC50) ranged from 0.16 to 0.34 mg/ml in organic extracts of the local ginger and 4.39–5.81 mg/ml in the hybrid ginger (Table 3). Non-significantly different low IC50 values were observed among the acetone, ethanol, and methanol extracts of the local ginger. Moderately high IC50 values were observed for organic extracts of hybrid ginger, while significantly high values were obtained in the aqueous extracts (8.93 and 6.44 mg/ml) of both the local and hybrid ginger (p˂0.05).

**Table 3:** IC50 (mg/ml) of ginger and garlic extracts in organic and aqueous extracts.

	Solvent				
Variety	Acetone	Ethanol	Methanol	Water	P value
Hybrid ginger	4.43 ± 0.10^b^	5.81 ± 0.13^c^	4.39 ± 0.05^b^	6.44 ± 0.01^d^	*0.001*
Local ginger	0.27 ± 0.05^a^	0.16 ± 0.01^a^	0.34 ± 0.05^a^	8.93 ± 0.01^e^	
Hybrid garlic	4.44 ± 0.019^c^	4.72 ± 0.002^e^	4.80 ± 0.006^e^	5.27 ± 0.002^f^	*0.001*
Local garlic	3.93 ± 0.013^a^	4.01 ± 0.028^b^	4.55 ± 0.031^d^	5.64 ± 0.045^g^	

Values = Mean ± SD (n = 3). Values with different superscripts along the row differ significantly for each spice.

This study suggests that local ginger has a higher free radical scavenging potential when compared to hybrid ginger and that organic solvents are more efficient in extracting compounds with strong free radical activity than water. Such a difference can be attributed to a difference in the genetic characteristics of the varieties and the polarity of the solvents.

Other studies report lower IC50 values in ethanolic extracts of fresh ginger rhizomes and dried ginger (65.82 and 14.69 μg/ml, respectively) [39]; petroleum ether and chloroform/methanol extracts of ginger rhizomes obtained from Sudan (8.29 and 29.87 μg/ml, respectively) [40]. However, our result is consistent with the range of 1C50 values reported for 16 ginger-like plants extracted with different solvents [23].

The IC50 of garlic extracts varied considerably between the organic solvent and aqueous extracts, as well as between local and hybrid varieties (p˂0.05). Acetone extracts of local and hybrid garlic (3.93 and 4.44 mg/ml) were the lowest; whereas water extracts of hybrid and local garlic (5.27 and 5.64 mg/ml) were the highest (Table 3). This shows that acetone is more effective than water at extracting free radical scavenging chemicals from garlic. The analyzed extracts' IC50 was within the range of 1.03–6.01 mg/ml published for garlic extracts [69]; and 4,376 to 8,540 g/ml recorded for garlic extracted with various solvents and techniques [70]. According to Ref. [10]; 50% ethanol extracts of spices were more powerful antioxidants than both ethanol alone and water extracts. In *Torilis leptophylla* assays, the half maximal inhibitory concentration of ethyl acetate fractions was found to be lower than that of methanol and aqueous fractions (62, 189, and 264 mg/ml, respectively) [45]; this is in agreement with our results.

#### 3.3.3 Relationship between IC50 and total phenolic content

**Fig. 3:**
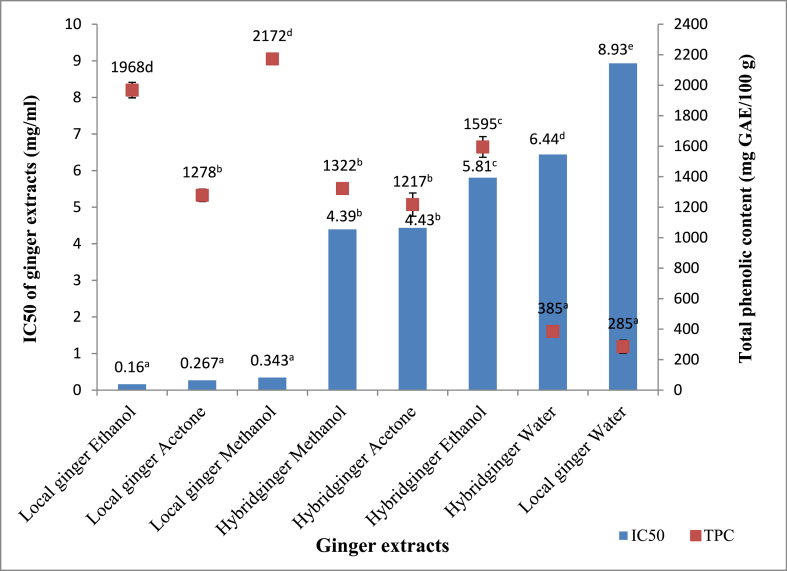
Relationship between IC 50 and Total phenolic content of ginger extracts. Values with the different superscript letters across the line are significantly different (p˂0.05).

The low IC50 of the DPPH assay was attributed to the high overall phenolic content of organic solvent extracts of local ginger (Fig. 3). However, there was a variation in variety, with methanol and acetone extracts of the hybrid ginger having significantly higher IC50 (p˂0.05) than ethanol extracts of the local ginger extract, although having statistically identical total phenolic content. The highest IC50 was associated with water extracts of both varieties with low overall phenolic content. The correlation between TPC, TFC, and IC50 of ginger extracts from aqueous and organic solvent extracts of the local and hybrid varieties was assessed using Pearson correlation. Results indicate a strong negative correlation between TPC and IC50 (r = −0.8011), p˂ 0.0001, TFC and IC50 (r = −0.6726), p˂0.05. This study suggests that ginger extract contains phytochemical constituents that are capable of donating hydrogen to a free radical to scavenge the potential damage.

Disparities in the IC50 of the solvent extracts with statistically similar TPC are attributed to the concentration of the individual phenolic compounds in the extracts. The antioxidant capabilities of phenolic compounds are dependent on the presence of OH groups in their chemical structure [23]. For instance, when ethanol extracts of ginger were compared to acetone, methanol, and water extracts of the same sample, ethanol extracts contained the largest amount of chlorogenic acid [7]. According to Ref. [38]; the content of gingerol and shaogoal, which are the main components responsible for antioxidant action in ginger, varied greatly with variety. Moreover, the IC50 of gingerol and shaogoal also differed [40].

**Fig. 4:**
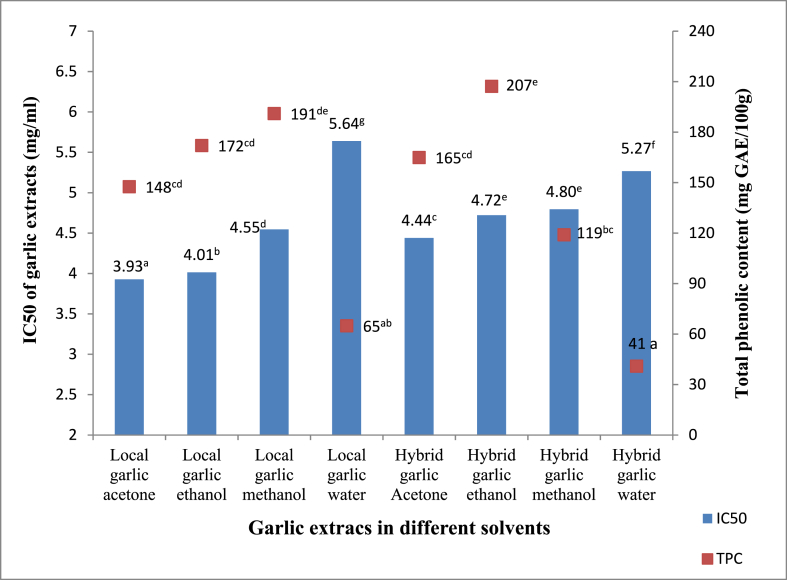
Relationship between IC50 and total phenolic content of garlic extracts. _Values with different superscript letters along the line are significantly different (p˂0.05)._

The relationship between the IC50 and TPC of garlic extracts is depicted in Fig. 4. The TPC of organic solvent extracts of the local garlic did not differ significantly, but the IC50 did (p˂ 0.05). Similarly, phenolic content was statistically comparable in methanol extracts of local garlic and ethanolic extracts of hybrid garlic with variable IC50. Acetone and water extracts of both varieties also had similar phenolic content but significantly different IC50. Pearson correlation showed that there was a strong negative correlation between the TPC of the extracts and the IC50 (r = −0.7804), (p˂0.0001), and between the TFC and IC50 (r = −0.5834), (p˂0.005). This means that higher TPC and TFC were associated with lower IC50 values, hence stronger antioxidant potential through the free radical scavenging mechanism.

The findings of this study showed that concentration of phenolic compounds and antioxidant activity were highly influenced by the solvent and variety. This result has previously been observed in a range of spices with varying amounts of specific phenolic compounds [10]; and different solvents with varying amounts of garlic bioactive components have been reported [71,70]. Moreover, the antioxidant activity of garlic components is shown to vary greatly, with each compound exhibiting different patterns of activity as free radical scavenging compounds [72,66,57].

## 4 Conclusion

The study concluded that ginger and garlic extracts are rich in phenolic compounds. In general, ginger extracts had more total phenolic, flavonoid, and vitamin C than garlic, and local ginger surpassed hybrid ginger. Organic solvents were more efficient than water in extracting phytochemicals with ethanol being more efficient compared to acetone, methanol. However, water was more efficient in extracting vitamin C compared to ethanol, methanol and acetone. The antioxidant activity of extracts assessed using the DPPH assays was strongly correlated to the total phenolic and flavonoid content. Ethanol, acetone and methanol extracts of the local ginger had high TPC and high antioxidant activities. The study proposes extracting phytochemicals using ethanol based on efficacy and safety. Quantifying the individual phenolic acids and flavonoids in ginger and garlic extracts is also recommended in order to promote their use as natural antioxidants.

## Author contribution statement

Jolly Oder Akullo: Conceived and designed the experiments; Performed the experiments; Analyzed and interpreted the data; Wrote the paper.

Beatrice N Kiage-Mokua, Dorothy Nakimbugwe: Conceived and designed the experiments; Analyzed and interpreted the data; Wrote the paper.

Jeremiah Ng’ang’a: Analyzed and interpreted the data; Wrote the paper.

John Kinyuru: Conceived and designed the experiments; Analyzed and interpreted the data; Contributed reagents, materials, analysis tools; Wrote the paper.

## Funding statement

Authors acknowledge funding from the German Academic Exchange services (DAAD) through the Regional Universities Forum for Capacity building in Agriculture, RUFORUM), In-Country/In-Region Scholarship Programme (1D:57429563); and the International Foundation for Science (IFS grant number: I-3-E- 6597–1).

## Data availability statement

Data included in article/supp. material/referenced in article.

## Declaration of competing interest

The authors declare that they have no known competing financial interests or personal relationships that could have appeared to influence the work reported in this paper.
